# Benefit of Adjuvant Radiotherapy for Local Control, Distant Metastasis, and Survival Outcomes in Patients with Localized Soft Tissue Sarcoma: Comparative Effectiveness Analysis of an Observational Cohort Study

**DOI:** 10.1245/s10434-017-6080-3

**Published:** 2017-09-11

**Authors:** Florian Posch, Richard Partl, Carmen Döller, Jakob M. Riedl, Maria Smolle, Lukas Leitner, Marko Bergovec, Bernadette Liegl-Atzwanger, Michael Stotz, Angelika Bezan, Armin Gerger, Martin Pichler, Karin S. Kapp, Herbert Stöger, Andreas Leithner, Joanna Szkandera

**Affiliations:** 1 Division of Oncology, Department of Internal Medicine, Medical University of Graz, Graz, Austria; 2 Comprehensive Cancer Center Graz, Medical University of Graz, Graz, Austria; 3 Department of Therapeutic Radiology and Oncology, Medical University of Graz, Graz, Austria; 4 Department of Orthopedics and Trauma Surgery, Medical University of Graz, Graz, Austria; 5 Institute of Pathology, Medical University of Graz, Graz, Austria; 6 Department of Experimental Therapeutics, University of Texas MD Anderson Cancer Center, Houston, TX USA

## Abstract

**Background:**

This study aimed to quantify the benefit of adjuvant radiotherapy (AXRT) for local control, distant metastasis, and long-term survival outcomes in patients with localized soft tissue sarcoma (STS).

**Methods:**

This single-center retrospective observational study enrolled 433 STS patients who underwent surgery with curative intent. An inverse probability of treatment-weighted (IPTW) analysis was implemented to account rigorously for imbalances in prognostic variables between the adjuvant treatment groups.

**Results:**

During a median follow-up period of 5.5 years, the study observed 38 local recurrences (9%), 73 occurrences of distant metastasis (17%), 63 STS-related deaths (15%), and 57 deaths from other causes (13%). As expected, patients receiving AXRT (*n* = 258, 60%) were more likely to have high-grade G3 tumors (*p* < 0.0001) than patients not receiving AXRT. A crude analysis showed that AXRT was not associated with improved recurrence-free survival [hazard ratio (HR) 1.00; 95% confidence interval (CI) 0.72–1.38; *p* = 0.98]. However, after IPTW, AXRT was associated with a 38% relative reduction in the risk of recurrence or death (HR 0.62; 95% CI 0.39–1.00; *p* = 0.05). This benefit was driven by a strong reduction in the risk of local recurrence (HR 0.42; 95% CI 0.19–0.91; *p* = 0.03), whereas the relative risk of distant metastasis (HR 0.69; 95% CI 0.39–1.25; *p* = 0.22) and overall survival (HR 0.76; 95% CI 0.44–1.30; *p* = 0.32) were only nonsignificantly in favor of AXRT. An exploratory analysis showed an overall survival benefit of AXRT for patients with high-grade G3 tumors (HR 0.51; 95% CI 0.33–0.78; *p* = 0.002). However, this finding may have been attributable to residual confounding.

**Conclusion:**

In this observational cohort, AXRT was associated with a 58% reduction in the relative risk of local recurrence. No consistent association between AXRT and lower risks of distant metastasis or death was observed.

**Electronic supplementary material:**

The online version of this article (doi:10.1245/s10434-017-6080-3) contains supplementary material, which is available to authorized users.

Adjuvant radiotherapy (AXRT) is an accepted treatment strategy for improving local control in patients with soft tissue sarcoma (STS) undergoing curative surgery.^1^,^2^ Besides reducing the risk of local recurrence, AXRT also may enable a more limited surgical approach and reduce the need for amputation.^3^ Nevertheless, the benefit of this intervention regarding nonlocal treatment outcomes such as distant metastasis and survival is currently ill-defined.^1^

Although randomized controlled trials would be optimal for quantifying the impact of AXRT on local and nonlocal outcomes for STS patients, patient accrual in this rare malignancy can be very difficult, leaving trials underpowered for detecting smaller effect sizes.^4^ For example, a pivotal randomized controlled trial of AXRT versus observation in STS found numerically higher overall survival estimates for patients treated with AXRT, but this difference did not reach statistical significance with the 141 enrolled patients.^1^

In contrast, observational cohort data for STS are copious^5^ but suffer from the drawback of nonrandom treatment assignment. The naïve estimation of AXRT treatment effects from such observational data harbors great potential for bias because clinicians generally assign patients with poor prognostic factors (e.g., high tumor grade or positive resection margins) to AXRT.^2^ This could lead to an underestimation of the “true” effect of AXRT. To address this problem, comparative effectiveness research methods have been developed.^6^ In this study, we aimed to quantify the contribution of AXRT to long-term outcomes for STS patients after surgical resection using a large observational study. We implemented an inverse probability of treatment-weighted (IPTW) analysis to account rigorously for nonrandom treatment assignment to AXRT.^7^

## Methods

### Study Population, Study Design, and Adjuvant Radiotherapy

This single-center, retrospective, observational study analyzed all 433 consecutive patients with histologically verified, localized STS of the extremity or the trunk who underwent surgery with curative intent between March 1998 and May 2015 at the Department of Orthopedics and Trauma Surgery, Medical University of Graz, Austria. This analysis population was drawn from our in-house STS database after exclusion of patients with evidence of distant metastasis at surgery (*n* = 14), patients who underwent neoadjuvant XRT (*n* = 5), patients with retroperitoneal STS (*n* = 8), and patients who were erroneously included (one with chondrosarcoma, two with surgery at the time of local recurrence).

Assignment to AXRT versus observation was based on a recommendation by our in-house orthopedic oncology tumor board for every patient. In general, the tumor board recommended AXRT for high-risk sarcomas identified by clinical criteria. Our in-house protocol for AXRT indication has remained consistent since 1998 and encompasses the following: no AXRT for liposarcoma grade G1 (also known as atypical lipomatous tumor [ALT]), no AXRT for other G1 tumors except in case of marginal resection without possibility of secondary resection, no AXRT for superficial/epifascial G2 tumors smaller than 5 cm, AXRT for all deep G2 tumors irrespective of size, AXRT for superficial/epifascial G2 tumors 5 cm in size or larger, and AXRT for all G3 tumors irrespective of size and localization. Standard radiation doses are 60 Gy for wide resections and 66 Gy for marginal resection.

The general surgical approach in our department during the study period was wide resection with a minimal margin of 1 mm (R0) for all STS patients except those with liposarcoma G1 (ALT), for which a marginal resection was considered to be adequate. Patients who underwent amputation (*n* = 29) were included in all analyses.

Baseline and outcome data were retrieved retrospectively from a prospectively maintained in-house electronic health care database, as previously reported.^8^,^9^ All events occurring during the follow-up period (local recurrence, distant metastasis, death due to STS vs death from other causes) were adjudicated by a medical oncologist (J.S.) and a medical student (J.R.). Data collection and analysis were approved by the local ethics committee (no. 29-091 ex 16/17).

### Statistical Analysis

All statistical analyses were performed using Stata (Windows version 14.0; Stata Corp., Houston, TX, USA). Distributional differences in baseline variables between the AXRT and observation groups were evaluated using Chi square and rank-sum tests. For each patient, the propensity score *e* was defined as the probability of receiving AXRT conditional on baseline covariates, and IPTW was defined as the inverse of the probability of receiving the treatment the patient received.^10^

Following best practice recommendations, we calculated the propensity score using a multivariable logistic regression model with augmentation including a broad set of variables irrespective of association with AXRT and clinical outcomes (Table S1).^6^,^11^ For this propensity score model, missing data of four variables (limb salvage, lymph node metastasis, postoperative complications, and unplanned excision) were imputed using a chained equations algorithm (Stata routine “mi impute chained”; full imputation model specified in Table S2).^12^

Between-group tests for differences were re-performed after weighting with the IPTW. The median follow-up was calculated with the inverse Kaplan–Meier estimator according to Schemper and Smith.^13^ Recurrence-free survival (RFS) and the risk of all-cause mortality were calculated with a (1–)Kaplan–Meier estimator, whereas competing risk cumulative incidence estimators according to Marubini and Valsecchi^14^ were implemented to calculate the risks of local recurrence, distant metastasis, overall recurrence, death due to STS, and death from other causes (Stata routine “stcompet” with death treated as the competing event).^15^,^16^ These estimators then were weighted with the IPTW for comparative effectiveness analysis.

Univariable unweighted and IPTW-weighted Cox proportional hazards models and Fine and Gray proportional sub-distribution hazards models were fitted for analyzing the association between AXRT and time-to-event end points, respectively.^17^ Potential interactions between AXRT and tumor grade were explored by fitting interactions in time-to-event analysis. The full analysis code is provided on request from the authors.

## Results

### Baseline Characteristics and Event Rates

The analysis included 433 patients (Table 1). At the time of primary surgery, the median age of the cohort was 62 years (range 19–93 years), and the distribution of gender was balanced (males: *n* = 221, 51%). Most patients had tumors featuring high-grade histology (G2/G3: *n* = 339 (78%), and approximately two thirds of the cohort had tumors larger than 5 cm (*n* = 301, 70%) and/or deep tumors (*n* = 277, 64%). Of 53 patients (12%) who received adjuvant chemotherapy,12 also received neoadjuvant chemotherapy courses.

**Table 1:** Baseline characteristics of the study population (*n* = 433)

Variable	Missing data^a^ *n* (%)	Overall (*n* = 433)	No AXRT (*n* = 175)	AXRT (*n* = 258)	*p* Value^b^	*p* Value^c^
Age at entry: years (range)	433 (0)	62 [47–73]	63 [49–75]	62 [45–73]	0.26	0.82
Female gender	433 (0)	212 (49)	89 (51)	123 (48)	0.52	0.61
Non-extremity location^d^	433 (0)	75 (17)	37 (21)	38 (15)	0.08	0.48
Deep tumor	433 (0)	277 (64)	108 (62)	169 (66)	0.42	0.63
Tumor size >5 cm	433 (0)	301 (70)	116 (66)	185 (72)	0.23	0.41
Resection margin not R0	433 (0)	39 (9)	26 (15)	13 (5)	<0.0001	0.36
Lymph node metastasis	426 (2)	10 (2)	2 (1)	8 (3)	0.18	0.86
Distant metastasis	433 (0)	0 (0)	0 (0)	0 (0)	N/A	N/A
Prior unplanned excision (i.e., “whoops procedure”)	399 (8)	154 (39)	48 (31)	106 (44)	0.008	0.58
Limb salvage	406 (6)	377 (93)	136 (86)	241 (98)	<0.0001	0.49
Histology	433 (0)	–	–	–	0.003	0.52
Liposarcoma	–	109 (25)	62 (35)	47 (18)	–	–
Myxofibrosarcoma	–	130 (30)	42 (24)	88 (34)	–	–
Leiomyosarcoma	–	43 (10)	16 (9)	27 (10)	–	–
Synovial sarcoma	–	30 (7)	9 (5)	21 (8)	–	–
MPNST	–	13 (3)	5 (3)	8 (3)	–	–
Other	–	108 (25)	41 (23)	67 (26)	–	–
Tumor grade	433 (0)	–	–	–	<0.0001	0.76
G1	–	94 (22)	81 (46)	13 (5)	–	–
G2	–	80 (18)	21 (12)	59 (23)	–	–
G3	–	259 (60)	73 (42)	186 (72)	–	–
AJCC stage 3	433 (0)	165 (38)	46 (26)	119 (46)	<0.0001	0.82
Postoperative complications^e^	405 (6)	105 (26)	33 (21)	72 (29)	0.06	0.15
(Neo)adjuvant chemotherapy	433 (0)	53 (12)	10 (6)	43 (17)	0.001	0.86

Distribution overall and by adjuvant radiotherapy. Continuous variables are summarized as medians with [25th percentile–75th percentile], whereas categorical variables are reported as absolute frequencies with (percentages)

*AXRT* adjuvant radiotherapy, *N*/*A* not applicable, *MPNST* malignant peripheral nerve sheath tumor, *AJCC* American Joint Committee on Cancer

^a^ No. of patients with observed values of the respective variable; % missing in round brackets

^b^ *p* Values for difference between patients without and with AXRT are from Pearson’s Chi square tests (categorical variables with expected cell counts ≥5), Fisher’s exact tests (categorical variables with expected cell counts <5), or Wilcoxon rank-sum tests (continuous variables)

^c^ *p* Values correspond to ^c^ but are from data after re-weighting with the inverse probability of treatment-weighted (IPTW) analysis

^d^ Non-extremity locations include thoracic/trunk (*n* = 70) and head/neck (*n* = 5)

^e^ Postoperative complications include wound healing deficit (*n* = 41), hematoma (*n* = 21), infection/abscess (*n* = 15), (flap) necrosis (*n* = 7), and others (*n* = 21)

After surgery, the patients were followed up for a median of 5.5 years, with 75% of the cohort followed up for more than 1.9 years and 25% for more than 9.9 years. The shortest follow-up period was 1 day for a patient lost to follow-up evaluation after hospital discharge, and the longest follow-up period was 16.8 years.

During the follow-up period, we observed 38 local recurrences (9%), and 73 distant metastases (17%). Both local recurrence and distant metastasis developed in 16 patients, for an overall recurrence in 95 patients (22%). Local recurrence developed for 1 (3.4%) of the 29 patients who underwent amputation.

Of the 121 patients (28%) who died, 63 had deaths attributable to STS, and 57 had deaths attributable to other causes. The cause of death was unclear for one patient. Overall, this corresponded to a 10-year cumulative risk of 13% (95% CI 9–17%) for local recurrence, 21% (95% CI 17–26%) for distant metastasis, 29% (95% CI 24–34%) for overall recurrence, 21% (95% CI 16–26%) for death due to STS, 21% (95% CI 16–27%) for death from other causes, and 43% (95% CI 37–50%) for death from any cause (Table 2, which also includes the corresponding 5-year results; Fig. S1). The 10-year overall survival (OS) rate was 57% (95% CI 50–64), and the RFS rate was 49% (95% CI 42–55).

**Table 2:** Outcomes at 5 and 10 years overall and by adjuvant radiotherapy (AXRT) status in unadjusted and inverse probability of treatment-weighted (IPTW) analysis

End point	Overall cohort (*n* = 433) % (95% CI)	No AXRT–unadjusted analysis (*n* = 175) % (95% CI)	AXRT–unadjusted analysis (*n* = 258) % (95% CI)	No AXRT–IPTW analysis (*n* = N/A) % (95% CI)	AXRT–IPTW analysis (*n* = N/A) % (95% CI)
5-Year risks					
Local recurrence	10 (7–13)	10 (6–16)	9 (6–14)	16 (12–20)	7 (4–9)
Distant metastasis	19 (15–23)	17 (11–24)	20 (15–26)	24 (20–29)	18 (15–22)
Recurrence-free survival	64 (58–69)	66 (58–74)	62 (55–69)	56 (N/A)	66 (N/A)
Death from any cause	26 (22–32)	24 (17–32)	28 (22–35)	33 (N/A)	24 (N/A)
10-Year risks					
Local recurrence	13 (9–17)	13 (7–20)	13 (8–19)	18 (13–22)	9 (6–12)
Distant metastasis	21 (17–26)	19 (12–26)	23 (17–29)	26 (22–30)	20 (16–24)
Recurrence-free survival	49 (42–55)	50 (40–60)	48 (39–56)	44 (N/A)	57 (N/A)
Death from any cause	43 (37–50)	62 (50–71)	55 (46–63)	43 (N/A)	39 (N/A)

Local recurrence and distant metastasis were estimated with competing risk analysis, recurrence-free survival with a Kaplan–Meier estimator, and death from any cause with a 1-Kaplan–Meier estimator

*CI* confidence interval, *N*/*A* not applicable (no. of patients in each study group is not meaningful in IPTW; *AXRT* Adjuvant radiotherapy, 95% CIs could not be estimated for recurrence-free survival and death from any cause in IPTW due to current lack of software implementation (Stata routine “sts generate” currently does not accommodate confidence interval estimation for weighted data)

### Quantifying the Benefit of AXRT: Crude Analysis

Adjuvant radiotherapy was applied to 258 patients (60%). The median AXRT dose was 60 Gy (25th–75th percentile: 60–60 Gy; range 20–68 Gy). Administration of AXRT was not performed for 21 (27%) of the 80 patients with G2 tumors or for 73 (28%) of the patients with G3 tumors.

**Fig. 1:**
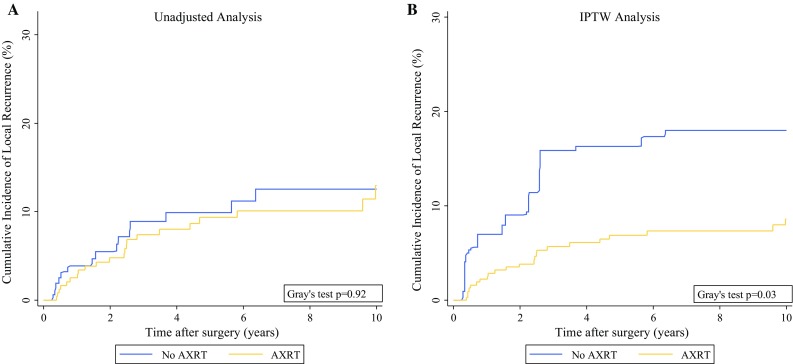
Cumulative incidence of local recurrence by adjuvant radiotherapy (AXRT) status. **a** Unadjusted analysis using a competing risk estimator with death from any cause as the competing event. **b** Inverse probability of treatment-weighted (IPTW) analysis using a competing risk estimator with death from any cause as the competing event

**Fig. 2:**
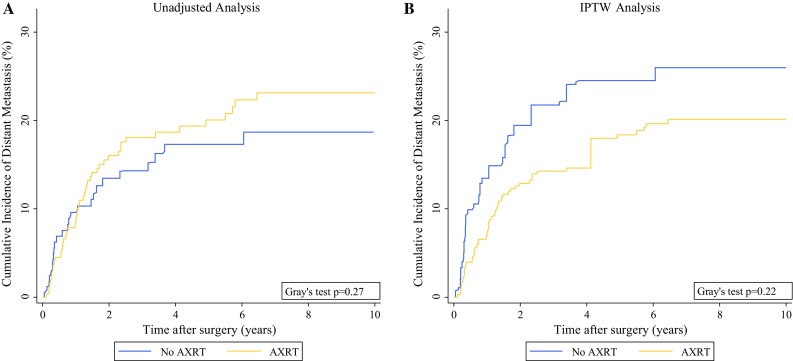
Cumulative incidence of distant metastasis by adjuvant radiotherapy (AXRT) status. **a** Unadjusted analysis using a competing risk estimator with death from any cause as the competing event. **b** Inverse probability of treatment-weighted (IPTW) analysis using a competing risk estimator with death from any cause as the competing event

**Fig. 3:**
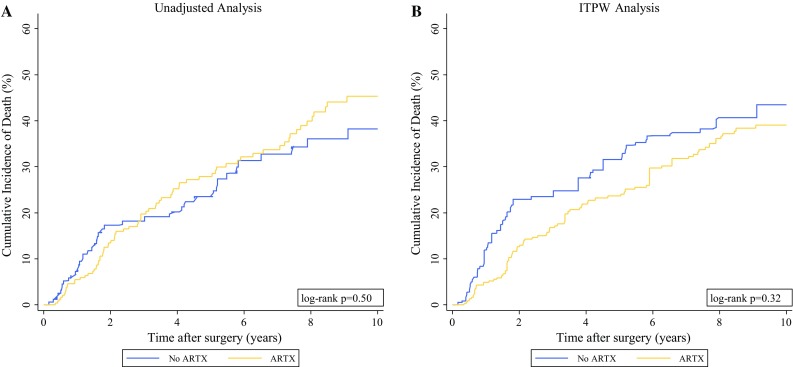
Cumulative incidence of death from any cause by adjuvant radiotherapy (AXRT) status. **a** Unadjusted analysis using a 1-Kaplan–Meier estimator. **b** Inverse probability of treatment-weighted (IPTW) analysis using an IPTW-weighted 1-Kaplan–Meier estimator

In a “crude” univariable time-to-event analysis, AXRT was not associated with RFS (HR 1.00; 95% CI 0.72–1.38; *p* = 0.98; 10-year RFS for patients with and without AXRT: 48% vs. 50%; Fig. S2A), OS (HR 1.14; 95% CI 0.78–1.66; *p* = 0.50), risk of local recurrence [sub-distribution hazard ratio (SHR) 0.92; 95% CI 0.48–1.76; *p* = 0.80], or risk of distant metastasis (SHR 1.20; 95% CI 0.74–1.95; *p* = 0.46). In detail, the 10-year risks for local recurrence, distant metastasis, and death from any cause were respectively 13, 23, and 45% for the patients who received AXRT and 13, 19, and 38% for the patients who did not receive AXRT (Figs. 1a, 2a, and 3a).

However, the patients who received AXRT had a significantly higher prevalence of adverse prognostic variables, such as high tumor grade, higher American Joint Committee on Cancer (AJCC) tumor stage, higher prevalence of prior unplanned excisions (“whoops procedures”), and more frequent administration of (neo-)adjuvant chemotherapy than the patients who did not receive AXRT (Table 1). This pattern is consistent with the expected non-random assignment of high-risk STS patients to AXRT by treating physicians.

### Quantifying the Benefit of AXRT: IPTW Analysis

We constructed IPTWs to account for the nonrandom treatment assignment to AXRT. This was performed by first predicting the propensity score, defined as the probability of being in the AXRT group conditional on covariates at baseline (Table S1, Fig. S3A). After transformation of the propensity score into the IPTW (Fig. S3B) and IPTW weighting of the data, statistically significant differences in baseline covariates between patients with and without AXRT did not prevail (Table 1).

Next, time-to-event analyses were re-performed after weighting for the IPTW. Here, AXRT was associated with a significant improvement in RFS (HR 0.62; 95% CI 0.39–1.00; *p* = 0.05). In detail, the 10-year RFS was 57% for the AXRT group and 44% for the patients not receiving AXRT (*p* = 0.05, log-rank; Fig. S2B). The difference in RFS between the XRT groups was driven by a strong reduction in the risk of local recurrence (SHR 0.42; 95% CI 0.19–0.91; *p* = 0.03), whereas the risks of distant metastasis (SHR 0.69; 95% CI 0.39–1.25; *p* = 0.22) and overall survival (HR 0.76; 95% CI 0.44–1.30; *p* = 0.32) were only nonsignificantly in favor of AXRT. The IPTW-adjusted 10-year risks of local recurrence, distant metastasis, and death were respectively 9, 20, and 39% for the patients who received AXRT and 18, 26, and 43% for the patients who did not receive AXRT (Figs. 1b, 2b, 3b). In a sensitivity analysis, we excluded one patient with an extremely high IPTW (29 times higher than the median IPTW; Fig. S3B). After exclusion of this potentially influential data point, the magnitude and strength of association for all the time-to-event estimates with AXRT prevailed (not shown).

### Exploratory Analysis of AXRT Benefit for Patients with G3 Tumors

All the patients with G3 tumors (*n* = 259) should have received AXRT according to our in-house AXRT protocol. However, only 186 of these patients (72%) actually received AXRT. After exclusion of the patients with amputation (*n* = 21), not receiving AXRT for G3 tumors was not significantly associated with postoperative complications (OR 0.61; 95% CI 0.26–1.41; *p* = 0.25).

The relative large proportion of patients with G3 tumors who did not receive AXRT allowed us to perform a hypothesis-generating post hoc analysis of AXRT effect modification by tumor grade via fitting interactions between AXRT and grade G3. In an unadjusted analysis, we observed a significant interaction between AXRT and grading for OS (*p* < 0.0001), with an OS benefit of AXRT for the patients with G3 tumors (HR for AXRT in G3 tumors, 0.51; 95% CI, 0.33–0.78; *p* = 0.002). This finding weakly prevailed after IPTW adjustment (corresponding HR 0.61; 95% CI 0.36–1.02; *p* = 0.06; *p* for interaction, 0.04). However, no consistent interaction between AXRT and G3 for distant metastasis risk was observed (unadjusted *p* for interaction, 0.05; IPTW-adjusted *p* for interaction, 0.41), with HRs only nonsignificantly in favor of AXRT (unadjusted HR 0.67; 95% CI 0.38–1.18; *p* = 0.16; IPTW-adjusted HR 0.63; 95% CI 0.35–1.14; *p* = 0.13).

## Discussion

This study used retrospective observational data and propensity score-based comparative effectiveness research methods to quantify the benefit of AXRT toward the risk of local recurrence, distant metastasis, and death among patients with STS after curative surgery. In a “naïve” analysis, AXRT was not associated with any of the study end points. However, after a rigorous accounting for imbalances in prognostic variables between treatment groups using IPTW, AXRT was associated with a strong reduction in the risk for local recurrence. Despite IPTW, we did not observe consistent evidence for a beneficial impact of AXRT on non-local end points such as distant metastasis and overall survival.

After surgery, patients with localized STS are at risk for local recurrence, distant metastasis, and death.^18^ Our hypothesis that AXRT may reduces distant metastasis risk and improves overall survival was motivated by data from both Gronchi et al.^20^ and our group,^19^ which demonstrate that local recurrence is a risk factor for distant metastasis and death among patients with localized STS after surgery. Although this finding is contradicted by a recent observational cohort study that did not find an impact of local recurrence on survival,^21^ we considered it clinically plausible that improved local control by AXRT may result in less dissemination of disease, and ultimately, improved survival. However, our current study of more than 400 STS patients did not yield evidence for this hypothesized non-local AXRT benefit. Although the direction of the AXRT “effect” estimates were in favor of AXRT for both distant metastasis risk and overall survival, these associations did not reach statistical significance with the numbers we had. Integrating these findings with the absence of other data in the literature showing an effect of AXRT beyond local recurrence risk reduction in STS,^1^ we consider the likelihood of an AXRT benefit on overall survival in the general population of STS patients to be limited.

An exception to this conclusion may be patients with high-grade G3 tumors. For this sub-population, we observed a weak association of AXRT with improved survival, and the hazard ratios (~ 0.6) suggested a potentially large magnitude of benefit. The validity of this finding is somewhat challenged by the absence of evidence for an AXRT benefit on distant metastasis risk in this sub-population. Although the event rate (and thus the statistical power for detecting relevant associations) was lower for distant metastasis than for survival in our study, distant metastasis is undoubtedly the major contributor to impaired long-term survival in STS.^19^ Hence, we reason that improvements in STS survival by AXRT must be mediated to a large degree by a reduction in metastasis risk. Our discrepant findings of a G3 subgroup benefit from AXRT between distant metastasis and survival may therefore indicate that the observed survival benefit from AXRT for G3 tumors may be more attributable to residual confounding not removed by the IPTW than to a true benefit of AXRT. Nevertheless, it can still be speculated that a larger study may have yielded statistically significant estimates of AXRT benefit for patients with G3 tumors. We therefore interpret this subgroup finding as hypothesis-generating.

Notably, a considerable proportion of patients in our cohort with G3 tumors did not receive AXRT despite an actual indication by our in-house AXRT protocol. We explored this discrepancy between indicated and eventually completed AXRT by analyzing postoperative complications, which may have hindered the application of AXRT. However, postoperative complications did not significantly predict for forgone AXRT in our study. Thus, it is likely that other factors yet to be defined hinder the indicated administration of AXRT for many patients with G3 tumors. In summary, this finding supports the careful speculation that neoadjuvant rather than adjuvant XRT may lead to a higher rate of adherence and completion of radiotherapy in this radio-sensitive cancer entity,^22^

In contrast to the systemic end points in this study, we found strong evidence that AXRT reduces the risk of local recurrence. The magnitude of effect in our study was approximately a 50% reduction in local recurrence risk with AXRT, which is highly consistent with previously published reports.^23^ This similarity between our data and these previous results supports the validity of our comparative effectiveness research strategy using propensity score methods. In summary, we believe that the currently available data represent evidence beyond reasonable doubt that AXRT improves local control for patients with localized STS.

Finally, we mention three limitations of this study. First, due to the study’s observational design, we could not rule out the presence of at least some residual confounding, which may have distorted our estimates of AXRT benefit. Importantly, the validity of the IPTW analysis depended on the assumption that the propensity score model is correctly specified and does not omit unmeasured confounders.^6^,^7^

Second, we excluded patients with neoadjuvant XRT because this patient subgroup was too small (*n* = 5) for a meaningful conclusion to be drawn in this study.

Third, we included a considerable proportion of G1 tumors. With exceptions, these patients are not routine candidates for AXRT.^2^ However, we kept these patients in the analysis to increase the variability of recurrence risks and sample size,^19^ which may ultimately lead to more robust estimates of AXRT benefit.

Within the limitations of this observational study, we concluded that our data provide strong support for the concept that AXRT improves local control for patients with localized STS after curative surgery. Regarding the magnitude of effect, we estimated that AXRT approximately halves the relative risk of local recurrence. However, we did not find consistent evidence for an impact of AXRT on non-local STS outcomes such as distant metastasis and death. In summary, these data can reassure sarcoma specialists that AXRT is an effective treatment for improving local control, but this improved local control does not necessarily translate into better systemic control and improved overall survival.

## Electronic supplementary material

Below is the link to the electronic supplementary material.

Supplementary material 1 (DOCX 38 kb)

## References

[1] Beane JD, Yang JC, White D, Steinberg SM, Rosenberg SA, Rudloff U (2014). Efficacy of adjuvant radiation therapy in the treatment of soft tissue sarcoma of the extremity: 20-year follow-up of a randomized prospective trial. Ann Surg Oncol..

[2] von Mehren M, Randall RL, Benjamin RS (2016). Soft Tissue Sarcoma, Version 2.2016, NCCN Clinical Practice Guidelines in Oncology. *JNCCN J Natl Comprehensive Cancer*. Network..

[3] Kneisl JS, Coleman MM, Raut CP (2014). Outcomes in the management of adult soft tissue sarcomas. J Surg Oncol..

[4] Yang JC, Chang AE, Baker AR (1998). Randomized prospective study of the benefit of adjuvant radiation therapy in the treatment of soft tissue sarcomas of the extremity. J Clin Oncol..

[5] Callegaro D, Miceli R, Bonvalot S (2016). Development and external validation of two nomograms to predict overall survival and occurrence of distant metastases in adults after surgical resection of localised soft-tissue sarcomas of the extremities: a retrospective analysis. Lancet Oncol..

[6] Austin PC, Stuart EA (2015). Moving towards best practice when using inverse probability of treatment weighting (IPTW) using the propensity score to estimate causal treatment effects in observational studies. Stat Med..

[7] Austin PC (2014). The use of propensity score methods with survival or time-to-event outcomes: reporting measures of effect similar to those used in randomized experiments. Stat Med..

[8] Kainhofer V, Smolle MA, Szkandera J (2016). The width of resection margins influences local recurrence in soft tissue sarcoma patients. Eur J Surg Oncol..

[9] Szkandera J, Gerger A, Liegl-Atzwanger B (2015). The derived neutrophil/lymphocyte ratio predicts poor clinical outcome in soft tissue sarcoma patients. Am J Surg..

[10] Austin PC (2011). An introduction to propensity score methods for reducing the effects of confounding in observational studies. Multivar Behav Res..

[11] White IR, Daniel R, Royston P (2010). Avoiding bias due to perfect prediction in multiple imputation of incomplete categorical variables. Computat Stat Data Anal..

[12] White IR, Royston P, Wood AM (2011). Multiple imputation using chained equations: issues and guidance for practice. Stat Med..

[13] Schemper M, Smith TL (1996). A note on quantifying follow-up in studies of failure time. Control Clin Trials..

[14] Marubini E, Valsecchi MG. Analysing survival data from clinical trials and observational studies. Chichester, England: Wiley; 2004. http://eu.wiley.com/WileyCDA/WileyTitle/productCd-0470093412.html.

[15] Panotopoulos J, Posch F, Alici B (2015). Hemoglobin, alkalic phosphatase, and C-reactive protein predict the outcome in patients with liposarcoma. J Orthop Res..

[16] Panotopoulos J, Posch F, Funovics PT (2016). Elevated serum creatinine and low albumin are associated with poor outcomes in patients with liposarcoma. J Orthop Res..

[17] Fine JP, Gray RJ (1999). A proportional hazards model for the subdistribution of a competing risk. J Am Stat Assoc..

[18] Patel SA, Royce TJ, Barysauskas CM, Thornton KA, Raut CP, Baldini EH (2017). Surveillance imaging patterns and outcomes following radiation therapy and radical resection for localized extremity and trunk soft tissue sarcoma. Ann Surg Oncol..

[19] Posch F, Leitner L, Bergovec M (2017). Can multistate modeling of local recurrence, distant metastasis, and death improve the prediction of outcome in patients with soft tissue sarcomas?. Clin Orthop Rel Res..

[20] Gronchi A, Lo Vullo S, Colombo C (2010). Extremity soft tissue sarcoma in a series of patients treated at a single institution: local control directly impacts survival. Ann Surg..

[21] Bonvalot S, Levy A, Terrier P (2017). Primary extremity soft tissue sarcomas: does local control impact survival?. Ann Surg Oncol..

[22] Davis AM, O’Sullivan B, Turcotte R (2005). Late radiation morbidity following randomization to preoperative versus postoperative radiotherapy in extremity soft tissue sarcoma. Radiother Oncol..

[23] Zhao RP, Yu XL, Zhang Z (2016). The efficacy of postoperative radiotherapy in localized primary soft tissue sarcoma treated with conservative surgery. Radiat Oncol London England..

