# Supplementary material for: Benefit of Adjuvant Radiotherapy for Local Control, Distant Metastasis, and Survival Outcomes in Patients with Localized Soft Tissue Sarcoma: Comparative Effectiveness Analysis of an Observational Cohort Study
Source: Ann Surg Oncol. 2017 Sep 11;25(3):776–83. doi: 10.1245/s10434-017-6080-3 (PMC5814515; doi:10.1245/s10434-017-6080-3)
Supplement: Supplementary file 1 — Supplementary material 1 (DOCX 38 kb) [file 10434_2017_6080_MOESM1_ESM.docx]

**Supplementary Tables (Online only)**

**Supplementary Table 1**

| **Supplementary Table 1. Conditional imputation models for 4 variables with missing data.** | | | | |
| --- | --- | --- | --- | --- |
|  | | | | |
| **Conditional imputation model** | **Number of observations to impute** | **Imputation model** | **Dependent variable** | **Independent variables** |
|  |  |  |  |  |
| #1 | 7 | Logistic regression | Lymph node metastasis (0/1) | - Postoperative complications - Prior unplanned excision - Sex - Age at surgery - Resection margin - Tumor grade - Histologic subtype - AJCC stage III - Deep location - Tumor size >5.0cm - (Neo-)adjuvant chemotherapy - Tumor Localization |
| #2 | 27 | Logistic regression | Limb Salvage (0/1) | - Lymph node metastasis - Postoperative complications - Prior unplanned excision - Sex - Age at surgery - Resection margin - Tumor grade - Histologic subtype - AJCC stage III - Deep location - Tumor size >5.0cm - (Neo-)adjuvant chemotherapy - Tumor Localization |
| #3 | 28 | Logistic regression | Postoperative complications (0/1) | - Lymph node metastasis - Limb salvage - Prior unplanned excision - Sex - Age at surgery - Resection margin - Tumor grade - Histologic subtype - AJCC stage III - Deep location - Tumor size >5.0cm - (Neo-)adjuvant chemotherapy - Tumor Localization |
| #4 | 34 | Logistic regression | Prior unplanned excision (0/1) | - Lymph node metastasis - Limb salvage - Postoperative complications - Sex - Age at surgery - Resection margin - Tumor grade - Histologic subtype - AJCC stage III - Deep location - Tumor size >5.0cm - (Neo-)adjuvant chemotherapy - Tumor Localization |

**Supplementary Table 2**

| **Supplementary Table 2. Mutivariable logistic regression model for predicting the propensity score.** | | | |
| --- | --- | --- | --- |
|  | | | |
| **Variable** | **Odds Ratio** | **95% CI** | **p** |
|  |  |  |  |
| Amputation | 0.12 | 0.04-0.32 | <0.0001 |
| Lymph node metastasis | 1.10 | 0.17-7.01 | 0.92 |
| Postoperative complications | 1.45 | 0.79-2.66 | 0.23 |
| Prior unplanned excision | 1.78 | 0.93-3.42 | 0.08 |
| Male sex | 0.89 | 0.54-1.47 | 0.66 |
| Age at surgery (per 1 year increase) | 0.98 | 0.97-1.00 | 0.05 |
| Resection margin R1 | 0.84 | 0.32-2.17 | 0.72 |
| Tumor grade G1 | Ref. | Ref. | Ref. |
| Tumor grade G2 | 21.27 | 8.36-54.10 | <0.0001 |
| Tumor grade G2 | 31.15 | 10.92-88.87 | <0.0001 |
| Histology - Fibrosarcoma | Ref. | Ref. | Ref. |
| Histology – Liposarcoma | 0.71 | 0.07-7.61 | 0.78 |
| Histology – Myxofibrosarcoma | 0.61 | 0.06-6.34 | 0.68 |
| Histology – Leimyosarcoma | 0.59 | 0.05-6.85 | 0.68 |
| Histology – Synovial sarcoma | 0.33 | 0.03-3.91 | 0.38 |
| Histology - MPNST | 0.15 | 0.01-2.10 | 0.16 |
| Histology - Other | 0.42 | 0.04-4.37 | 0.47 |
| AJCC stage III | 0.81 | 0.32-2.09 | 0.67 |
| Deep tumor location | 0.85 | 0.48-1.50 | 0.57 |
| Tumor size > 5.0 cm | 2.43 | 1.07-5.52 | 0.04 |
| (Neo-)adjuvant chemotherapy | 1.78 | 0.68-4.65 | 0.24 |
| Localization – Upper extremity | Ref. | Ref. | Ref. |
| Localization – Lower extremity | 0.66 | 0.34-1.28 | 0.22 |
| Localization – Thoracic / Trunk | 0.49 | 0.21-1.11 | 0.09 |
| Localization – Head / Neck | 0.44 | 0.04-4.29 | 0.48 |

# Supplementary Table legends (Online only)

# Supplementary Table 1. Conditional imputation models for 4 variables with missing data. Multiple imputation was performed using augmented logistic regression. Five imputation datasets were generated.

# Supplementary Table 2. Multivariable logistic regression model for predicting the propensity score. In this model, adjuvant radiotherapy (0=no AXRT, 1=AXRT) was the dependent variable. Estimation results represent odds ratios with 95% confidence intervals and p-values for predicting treatment assignment.

**Supplementary Figures (Online only)**

**Supplementary Figure 1**

**Supplementary Figure 2**

**Supplementary Figure 3**

# Supplementary Figure legends (Online only)

**Supplementary Figure 1. Cumulative risks of different clinical outcomes in the overall study population (n=433).** The risk of death-from-any-cause was estimated with a 1-Kaplan-Meier estimator, whereas all other endpoint risks were computed with competing risk cumulative incidence estimators treating death-from-any-cause as the competing event (exceptions: Death-from-STS: Death-from-other-causes as the competing event; Death-from-other-causes: Death-from-STS as the competing event).

**Supplementary Figure 2. Figure 3. Recurrence-free survival (RFS) by AXRT status.** Recurrence-free-survival was defined as the time-to-death-from-any-cause or local recurrence of distant metastasis, whatever occurred first. (3A) Unadjusted analysis using a Kaplan-Meier estimator. (3B) IPTW analysis using an IPTW-weighted Kaplan-Meier estimator.

**Supplementary Figure 3. Histograms of the Propensity Score and the IPTW.** (3A) The propensity score can range from 0 to 1. Multiply by 100 to obtain probabilities (in percent) of having received AXRT. Median propensity score was 0.71 [25^th^-75^th^ percentile: 0.34-0.82]. (3B) The IPTW was defined as the inverse of the probability of receiving the treatment that the patient actually received (i.e. the so-called “average treatment effect on the treated (ATT)”). Median IPTW was 1.3 [25^th^-75^th^ percentile: 1.2-1.7]. One outlying value is present in the IPTW (value: 37.6, i.e. 22 times higher than the 75^th^ percentile of the IPTW distribution).
